# Differential response of adipose tissue gene and protein expressions to 4‐ and 8‐week administration of *β*‐guanidinopropionic acid in mice

**DOI:** 10.14814/phy2.13616

**Published:** 2018-03-07

**Authors:** Hisashi Kato, Shinya Masuda, Tomotaka Ohira, Luna Ohira, Hisashi Takakura, Yoshinobu Ohira, Tetsuya Izawa

**Affiliations:** ^1^ Faculty and Graduate School of Health and Sports Science Doshisha University Kyotanabe Japan; ^2^ Chiben Gakuen Nara College Kashiba Japan; ^3^ Faculty of Health and Well‐being Kansai University Sakai Japan; ^4^ Research Center for Adipocyte and Muscle Science Doshisha University Kyotanabe Japan; ^5^ Research Center for Space Medical Science Doshisha University Kyotanabe Japan; ^6^ Graduate School of Medicine Osaka University Toyonaka Japan

**Keywords:** Brown adipose tissue, PGC‐1*α*, PPAR*α*, white adipose tissue

## Abstract

*β*‐Guanidinopropionic acid (*β*‐GPA) feeding inhibits growth‐associated gain of body mass. It remains unknown, however, whether and how *β*‐GPA feeding affects growth‐associated increase in white adipose tissue (WAT) mass. We examined the effects of 4‐ and 8‐week *β*‐GPA feeding on serum myostatin levels and expression of genes and proteins related to adipogenesis, lipolysis, and liposynthesis in epididymal WAT (eWAT) and brown adipose tissue (BAT) in 3‐week‐old, juvenile male mice. Body, eWAT, and muscle weights were significantly lower in *β*‐GPA‐fed mice than in controls after feeding. Four‐ but not 8‐week‐*β*‐GPA feeding increased the serum myostatin level. Incubation of C2C12 myotubes with *β*‐GPA (1 mM) significantly promoted myostatin mRNA expression. The protein expression of peroxisome proliferator‐activated receptor gamma coactivator 1 *α* (PGC‐1*α*) and peroxisome proliferator‐activated receptor *α* (PPAR *α*) was up‐regulated in GPAF eWAT at week 4, but down‐regulated at week 8. There was no significant difference in the protein expression of adipocyte triglyceride lipase (ATGL), hormone‐sensitive lipase (HSL), fatty acid synthase (FAS), and acetyl‐CoA carboxylase (ACC) between groups in eWAT. In BAT, no significant difference was found in the protein expression of PGC‐1*α*, PPAR *α*, ATGL, and HSL between *β*‐GPA‐fed and control mice, whereas that of FAS and ACC was significantly lower in *β*‐GPA‐fed mice at week 8. Uncoupling protein 1 was expressed higher in *β*‐GPA‐fed mice both at weeks 4 and 8 than that in controls. Thus, the mechanism by which *β*‐GPA feeding in early juvenile mice inhibits growth‐associated increase in eWAT mass may differ between early and later periods of growth.

## Introduction

*β*‐Guanidinopropionic acid (*β*‐GPA) has a molecular structure similar to that of creatine, and supplementation with *β*‐GPA decreases the cellular content of both creatine and phosphocreatine (Ohira et al. 2003; Oudman et al. 2013). The creatine kinase/phosphocreatine system is used as an energy shuttle between mitochondrial ATP production and cytosolic ATP consumption in cells, and plays a pivotal role in maintaining cellular energy homeostasis (Wallimann and Hemmer 1994; Schlattner et al. 2006). Therefore, the inhibition of creatine activity by *β*‐GPA alters cellular energy metabolism and, as a consequence, affects overall body composition, including body weight and skeletal muscle mass, in animals (Wakatsuki et al. 1994, 1995; Levine et al. 1996; Oudman et al. 2013). A recent study (Baumgarner et al. 2015) examined the mechanisms underlying the inhibition of skeletal muscle growth during long‐term feeding of *β*‐GPA in young juvenile mice; this study showed that several anabolic and catabolic signals are differentially controlled between early and later phases of the feeding period. It remains unknown, however, whether and how *β*‐GPA feeding affects growth‐associated increase in white adipose tissue (WAT) mass in young juvenile mice.

The different responses of anabolic and catabolic signals in skeletal muscle between early and later phases during long‐term *β*‐GPA feeding (Baumgarner et al. 2015) suggest the possibility that circulating and/or secreting levels of myostatin may differ between these two phases. Myostatin, a protein that is produced and released predominantly by skeletal muscle (Mcpherron et al. 1997), is well known as a potent negative regulator of skeletal muscle mass (Mcpherron et al. 1997; Ríos et al. 2002; Snijders et al. 2015) and has an antagonistic effect on anabolic signals in this type of muscle (Burnett et al. 1998; Trendelenburg et al. 2009; Egerman and Glass 2014). Myostatin has also been shown to inhibit both the adipogenic differentiation of white adipocytes in vitro (Rebbapragada et al. 2003; Li et al. 2011; Kim et al. 2012) and fat accumulation in 3T3‐L1 cells by inhibiting the expression of critical lipogenic enzymes and promoting the expression of lipolytic enzymes (Zhu et al. 2015). Therefore, we hypothesized that adipogenic differentiation and fat metabolism, which affect growth‐associated increase in WAT mass, are differently regulated between early and late phases of long‐term *β*‐GPA feeding in concert with the change in serum myostatin level. In this study, we verified this hypothesis using young juvenile mice. In addition, we examined the effects of *β*‐GPA feeding on molecules involved in fat metabolism in brown adipose tissue (BAT), which is well known for controlling energy homeostasis in the whole body.

## Materials and Methods

### Experimental design and animal care

**Figure 1:**
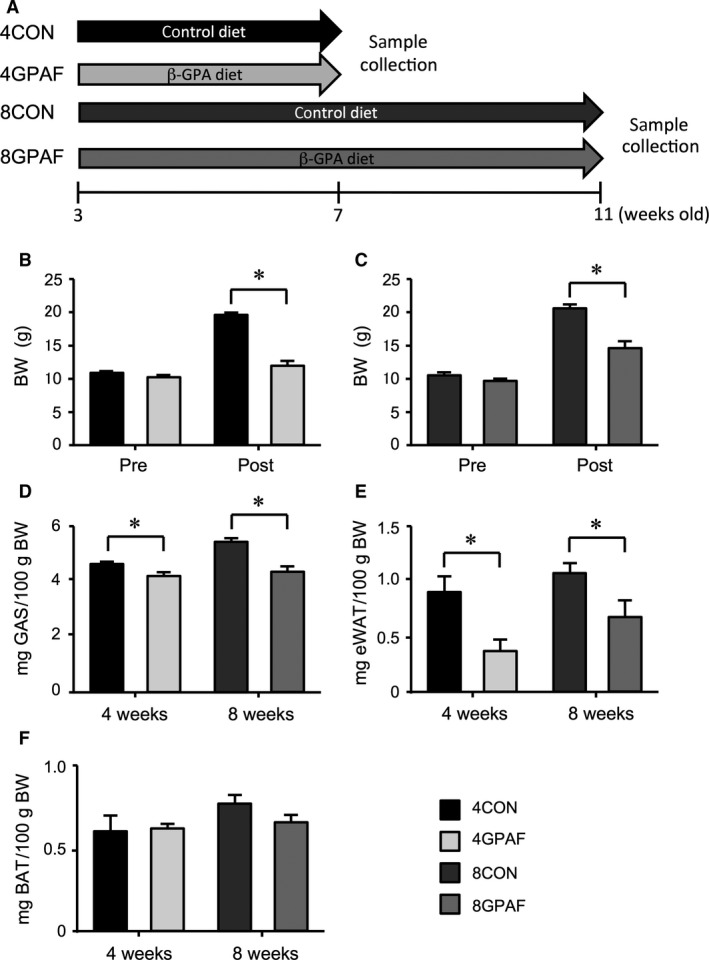
Effects of *β*‐GPA feeding on body composition (A) Schematic presentation of *β*‐GPA feeding to early juvenile mice: 4GPAF and 8‐week *β*‐GPA‐fed group (8GPAF) were fed powdered diet containing 1% *β*‐GPA, whereas control groups for the corresponding feeding periods (4CON and 8CON, respectively) were fed powdered diet without *β*‐GPA. The mice were pair‐fed throughout the feeding period. (B, C) BW prior to and following the *β*‐GPA‐feeding period for 4 (B) and 8 (C) weeks. (D) GAS weight relative to BW (E) eWAT weight relative to BW (F). BAT weight relative to BW. Black and gray bars indicate CON and GPAF, respectively. Values are the means ± SEM; *significant difference, *P* < 0.05; abbreviations: *β*‐GPA,* β*‐guanidinopropionic acid; 4GPAF, 4‐week *β*‐GPA‐fed group; 8GPAF, 8‐week *β*‐GPA‐fed group; 4CON, 4‐week control group; 8CON, 4‐week control group; Pre, before *β*‐GPA feeding; Post, after *β*‐GPA feeding; BW, body weight; GAS, gastrocnemius muscle; eWAT, epididymal white adipose tissue; BAT, brown adipose tissue.

The experimental procedures were conducted in accordance with the Guide for the Care and Use of Laboratory Animals of the Japanese Physiological Society. This study was also approved by the Committee on Animal Care and Use at the Graduate School of Medicine, Osaka University. Newly weaned male C57BL/6J mice aged 3 weeks were randomly divided into four groups (*n* = 5 for each group) according to diet and feeding period: 4‐week *β*‐GPA‐fed group (4GPAF), 8‐week *β*‐GPA‐fed group (8GPAF), and control groups for the corresponding feeding periods (4CON and 8CON, respectively). The mice in the control group were fed powdered diet (CE‐2, Nihon CLEA, Tokyo, Japan) for 4 (4CON) or 8 weeks (8CON). The mice in the GPAF groups were fed the same powdered diet but containing 1% *β*‐GPA, as described previously (Wakatsuki et al. 1994, 1996; Yamashita et al. 1995). They were pair‐fed and water was supplied ad libitum. The food was completely consumed within approximately 12 h, and the amount of food was gradually increased according to the growth during the first 2 weeks; approximately 3.3 g food per mouse was supplied daily thereafter. At the 4th or the 8th week, mice were sacrificed under anesthesia with *i.p*. injection of sodium pentobarbital (50 mg/kg) and blood samples were collected for measurements of serum myostatin levels after measurement of body weight. Then, epididymal WAT (eWAT), interscapular BAT, and gastrocnemius muscle in both hind limbs were collected from each mouse (Fig. 1A).

### Measurement of serum myostatin concentration

Serum myostatin concentration was measured using a GDF‐8/Myostatin ELISA kit (R&D Systems, Minneapolis, MN, USA) according to the manufacturer's instructions. Measurements were performed in duplicate for each sample.

### Cell culture

C2C12 myoblast cells were kindly provided by Dr. Takeshi Hashimoto (Ritsumeikan University). C2C12 cells were seeded at 25,000 cells/cm^2^ in growth medium. After 24 h, the cells were differentiated in Dulbecco's modified Eagle's medium (DMEM) containing 2% horse serum and 1% penicillin‐streptomycin (PS) for 96 h to allow full differentiation. Then, *β*‐GPA was added to the medium at a final concentration of 1 mmol/L. After 24 h, the C2C12 cells were harvested for analyses.

3T3‐L1 cells were seeded at 5000 cells/cm^2^ and allowed to proliferate in growth media containing 10% fetal bovine serum (FBS) and 1% PS for 72 h. Then, adipogenic differentiation was initiated by exchanging the growth media with DMEM containing 10% FBS, 1 *μ*mol/L dexamethasone, 10 *μ*g/mL insulin, 0.5 mmol/L 3‐isobutyl‐1‐methylxanthine, and 1% PS (day 0). On day 2, the media was exchanged with DMEM containing 10% FBS, 10 *μ*g/mL insulin, and 1% PS. On day 4, the cells were maintained in growth media, which was refreshed every 48 h. Treatment with recombinant human myostatin, at a final concentration of 10 nmol/L, was initiated at the onset of adipogenic differentiation (day 0); the recombinant myostatin was refreshed every 48 h until cells were harvested. On day 8, 3T3‐L1 adipocytes were harvested for analysis.

### Real‐time polymerase chain reaction (PCR)

Total RNA was extracted from eWAT, BAT, and cultured C2C12 cells, using ISOGEN II (Nippon Gene, Tokyo, Japan) according to manufacturer's protocol. cDNAs were synthesized from 1 *μ*g of total RNA, using the PrimeScript II 1st strand cDNA Synthesis Kit (TaKaRa Bio). Primers sequences are shown in Table 1. Real‐time PCR was performed in duplicate using KAPA SYBR FAST qPCR Kit Master Mix ABI Prism (KAPA BIO, Wilmington, MA, USA) on Applied Biosystems StepOne Real‐Time PCR System (Applied Biosystems, Waltham, MA, USA). The amplification protocol was as follows: stage 1 – denaturation for 20 sec at 95°C; stage 2–40 cycles consisting of denaturation for 3 sec at 95°C and annealing for 30 sec at 60°C; stage 3 – melt curve analysis. Gene expression was quantified using the standard curve method and normalized to that of ribosomal protein S18 (*Rps18*) (for tissues) or *β*‐actin (*Actb*) (for cells).

**Table 1:** Primer sequences

Primers for mouse mRNA		
Gene name	Forward (5′–3′)	Reverse (5′–3′)
Acc	GAAGTCAGAGCCACGGCACA	GGCAATCTCCAGTTCAAGCCAGTC
Atgl	CACTTTAGCTCCAAGGATGA	TGGTTCAGTAGGCCATTCCT
Cebp*α*	ACATCAGCGCCTACATCGACC	TTGGCCTTCTCCTGCTGTCG
Cebp*β*	ACCGGGTTTCGGGACTTGA	CCCGCAGGAACATCTTTAAGTGA
Fabp4	ACCGCAGACGACAGGAA	CTCATGCCCTTTCATAAAC
Fas	CCCAGCCCATAAGAGTTACA	ATCGGGAAGTCAGCACAA
Hsl	GGCAGTGGTGTGTAACTAGGATTG	ATCCATGCTGTGTGAGAACGC
Mstn	CAGCCTGAATCCAACTTAGG	TCGCAGTCAAGCCCAAAGTC
Ppara	GCAAGAGAATCCACGAAGCC	CGTCTTCTCGGCCATACACA
Ppar*γ*	GGAGCCTAAGTTTGAGTTTGCTGTG	TGCAGCAGGTTGTCTTGGATG
Pgc1a	CACCGTAAATCTGCGGGATG	TATCCATTCTCAAGAGCAGCGAAAG
Slc2a4	GTGGGTTGTGGCAGTGAGTC	CCCCATCGTCAGAGCCGATC
Rps18	TTCTGGCCAACGGTCTAGACAAC	CCAGTGGTCTTGGTGTGCTGA
Actb	TCAGCAAGCAGGAGTACGATG	AGCTCAGTAACAGTCCGCCTA

Acc, acetyl‐CoA carboxylase; Atgl, adipocyte triglyceride lipase; Cebp*α*, CCAAT/enhancer‐binding protein *α*; Cebp*β*, CCAAT/enhancer‐binding protein *β*; Fabp4, fatty acid binding protein 4; Fas, fatty acid synthase; Hsl, hormone‐sensitive lipase; Mstn, myostatin; Ppar*α*, peroxisome proliferator‐activated receptor *α*; Ppar*γ*, peroxisome proliferator‐activated receptor *γ*; Pgc1*α*, PPAR*γ* coactivator 1*α*; Slc2a4, glucose transporter 4; Rps18, Ribosomal Protein S18; Actb, actin beta.

### Western blotting

Western blotting was performed as described previously (Kato et al. 2015; Tanaka et al. 2015) with a slight modification: eWAT and BAT samples were homogenized in lysis buffer (20 mmol/L Tris‐HCl pH 7.4, 1% Triton‐X100, 50 mmol/L sodium chloride, 1 mmol/L ethylenediaminetetraacetic acid, 250 mmol/L sucrose, 50 mmol/L sodium fluoride, 5 mmol/L sodium diphosphate, 1 mmol/L dithiothreitol, 1 mmol/L sodium orthovanadate, 4 mg/mL leupeptin, 1 mg/mL pepstain A, and 0.5 mmol/L phenylmethylsulfonyl fluoride). The homogenate was incubated on ice for 15 min and centrifuged twice at 14,000*g* for 15 min at 4°C. Then, clear supernatant was recovered.

Protein concentration was quantified using Bio‐Rad Protein Assay (Bio‐Rad, Hercules, CA, USA); then, each sample was mixed with Laemmli sample buffer and heated for 2 min at 95°C. After separation by sodium dodecyl sulfate‐polyacrylamide gel electrophoresis, proteins were transferred to polyvinylidene difluoride membranes, which were blocked with Tris‐buffered saline (TBS) containing 0.1% Tween‐20 (TBS‐T) and 5% skim milk overnight at 4°C. Membranes were then probed for 3 h at room temperature in TBS‐T containing 5% bovine serum albumin and 1:500 dilutions of specific antibodies against peroxisome proliferator‐activated receptor (PPAR)*γ* (Santa Cruz Biotechnology, Santa Cruz, CA, USA), hormone‐sensitive lipase (HSL) (Cell Signaling Technology, Danvers, MA, USA), adipocyte triglyceride lipase (ATGL) (Cell Signaling Technology), fatty acid synthase (FAS) (Cell Signaling Technology), acetyl‐CoA carboxylase (ACC) (Cell Signaling Technology), PPAR*γ* coactivator 1*α* (PGC‐1*α*) (Merck Millipore, Billerica, MA, USA), uncoupling protein 1 (UCP1) (Abcam, Cambridge, UK) or *β*‐actin (Abcam). Subsequently, membranes were labeled with 1:5000 dilutions of anti‐rabbit or anti‐mouse immunoglobulin G (GE Healthcare, Buckinghamshire, UK) at room temperature for 60 min. Bands were visualized using ECL Prime system (GE Healthcare) and quantified on ChemiDoc™ MP system (Bio‐Rad, Hercules, CA, USA). Protein levels were normalized to that of *β*‐actin.

### Statistical analysis

Values are expressed as the means ± standard error of the mean (SEM). A paired two‐tailed student *t*‐test was performed for comparison of body weight between pre‐ and postfeeding periods, and an unpaired two‐tailed student *t*‐test was performed for comparison of the other parameters. *P *<* *0.05 was considered to represent a statistically significant difference.

## Results

Body weights did not differ significantly between the *β*‐GPA‐fed and control groups prior to the feeding period; however, these were significantly lower in the 4GPAF and 8GPAF groups than in the 4CON and 8CON groups at the end of the feeding period, by 39% and 30%, respectively, (Figs. 1B and C). Gastrocnemius muscle weight, normalized to body weight, was significantly less in 4GPAF and 8GPAF than in 4CON and 8CON, by 10% and 20%, respectively, (Fig. 1D). eWAT weight, normalized to body weight, was also significantly lower in 4GPAF and 8GPAF than in 4CON and 8CON, by 59% and 37%, respectively, (Fig. 1E). Conversely, BAT weight, normalized to body weight, was not significantly different between groups (Fig. 1F).

**Figure 2:**
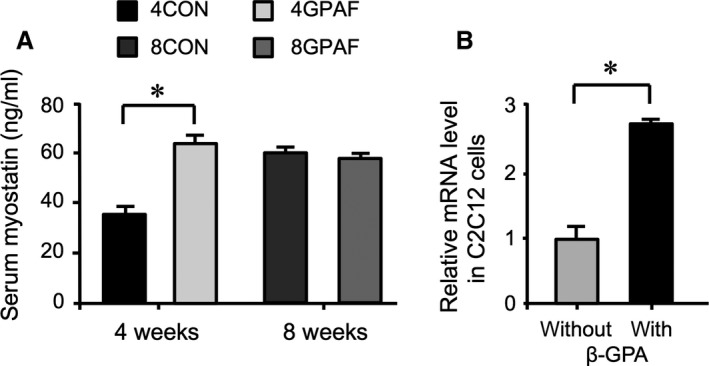
Effects of *β*‐GPA feeding on serum myostatin levels in mice and *β*‐GPA‐induced increase in Mstn mRNA expression (A) Serum myostatin concentration in mice fed with *β*‐GPA (B) Expression of Mstn in C2C12 myotubes treated with *β*‐GPA for 24 h; mRNA levels were normalized to those of Rps18, and the value in the presence of *β*‐GPA was related to the value in the absence of *β*‐GPA (set to 1). Values are the means ± SEM; * significant difference, *P* < 0.05; abbreviations: Mstn, myostatin; others are as defined in the legend of Figure 1.

An elevated serum myostatin concentration was observed in 4GPAF compared with that in 4CON (Fig. 2A); however, the serum myostatin level was not significantly different between 8GPAF and 8CON. We examined the direct effect of *β*‐GPA (1 mmol/L) on the expression of myostatin (*Mstn*) in C2C12 myotubes in vitro. As shown in Figure 2B, the mRNA expression of *Mstn* in the C2C12 myotubes was significantly elevated in the presence of *β*‐GPA.

**Figure 3:**
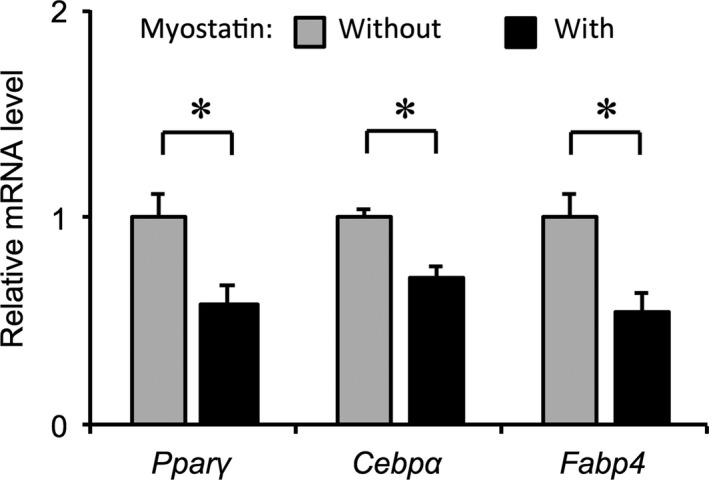
Expression of genes involved in adipogenesis (Ppar*γ*, Cebp*α*, and Fabp4 mRNA) in 3T3‐L1 cells treated with 10 nmol/L myostatin during adipogenic differentiation; mRNA levels were normalized to those of Rps18, and the values for *β*‐GPA‐fed mice were related to the values for each respective control (set to 1).; values are the means ± SEM; *significant difference, *P* < 0.05; abbreviations are as defined in the legend of Table 1.

The long‐term in vitro effect of recombinant myostatin protein on the expression of marker genes for adipogenesis was examined in cultured 3T3‐L1 cells during differentiation, as shown in Figure 3. The expression of adipogenesis marker genes, *Pparγ*, CCAAT/enhancer‐binding protein (*Cebp*) *α*, and fatty acid binding protein 4 (*Fabp4*), was considerably lower in myostatin‐treated 3T3‐L1 cells compared with that in nontreated cells.

**Figure 4:**
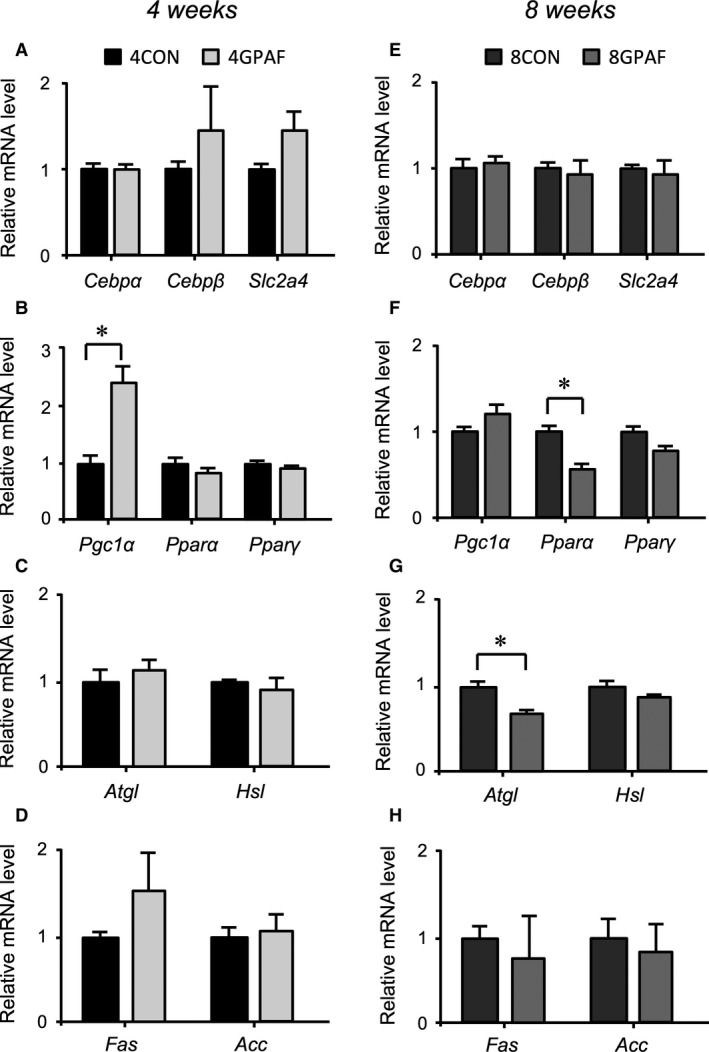
Alteration in the mRNA expression of genes related to adipogenesis and fat metabolism in eWAT: mRNA levels were normalized to those of Rps18, and the values for *β*‐GPA‐fed mice were related to the values for each respective control (set to 1). (A, E) Expression of Cebp*α*, Cebp*β*, and Slc2a4 mRNA at week 4 (A) and 8 (E) (B, F) Expression of Pgc1*α*, Ppar*α*, and Ppar*γ* mRNA at week 4 (B) and 8 (F) (C, G) Expression of Atgl and Hsl mRNA at week 4 (C) and 8 (G) (D, H) Expression of Fas and Acc mRNA at week 4 (D) and 8 (H); values are the means ± SEM; *significant difference, *P* < 0.05; abbreviations are defined in the legends of Table 1 and Figure 1.

We next examined the effects of *β*‐GPA feeding on the mRNA expression of genes related to adipogenesis (*Cebpα*,* Cebpβ*,* Pparγ*), glucose transport (*Slc2a4*), mitochondrial oxidation (*Pgc1α* and *Pparα*), lipolysis (*Atgl* and *Hsl*), and lipogenesis (*Fas* and *Acc*) in eWAT. As shown in Figure 4A and E, there was no significant difference in the mRNA expression of marker genes for adipogenesis, *Cebpα* and *Cebpβ*, between *β*‐GPA‐fed and control groups. At the 4‐week time point of *β*‐GPA feeding, the expression of *Pgc1α* was significantly higher in 4GPAF than in 4CON (Fig. 4B). Conversely, at the 8‐week time point of *β*‐GPA feeding, the mRNA expression of *Pparα* was significantly lower in 8GPAF than in 8CON (Fig. 4F). In addition, the mRNA expression of *Atgl* was significantly lower in 8GPAF than in 8CON (Fig. 4G). In comparison, the mRNA expression levels of *Hsl*,* Fas*, or *Acc* were not significantly different between the *β*‐GPA‐fed and control groups (Fig. 4C, D, G and H). The mRNA expression of *Ucp1* could not be detected in eWAT from any of the groups.

**Figure 5:**
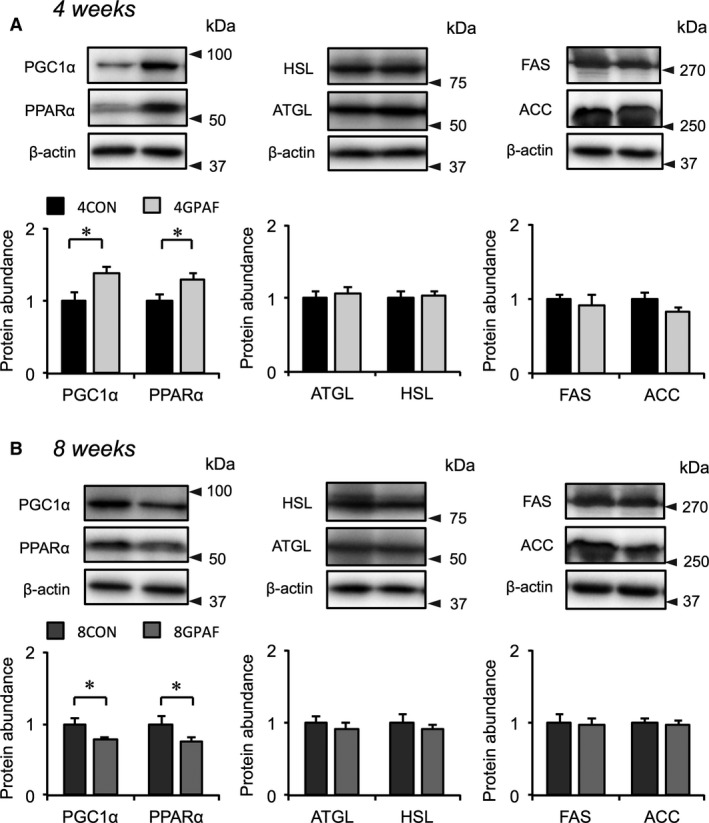
Alterations in the protein expression of PGC‐1*α*, PPAR *α*, ATGL, HSL, FAS, and ACC in eWAT at week 4 (A) and 8 (B): the levels of protein expression were normalized to that of *β*‐actin, and the values for GPA‐fed mice were related to the value for each respective control (set to 1). Representative western blotting bands are shown above the corresponding bars. Values are the means ± SEM; *significant difference, *P* < 0.05; abbreviations are defined in the legends of Table 1 and Figure 1.

With reference to the results shown in Figure 4, the expression of some of the proteins was examined. As shown in Figure 5A, the protein expression of PGC‐1*α* and PPAR*α* was significantly higher in 4GPAF than in 4CON. Conversely, the expression of these proteins was significantly lower in 8GPAF than in 8CON (Fig. 5B). Thus, the effects of *β*‐GPA feeding on the expression of these proteins were quite different between the 4‐week and the 8‐week time points. There was no significant difference in the protein expression of ATGL, HSL, FAS, and ACC between the *β*‐GPA‐fed and control groups.

**Figure 6:**
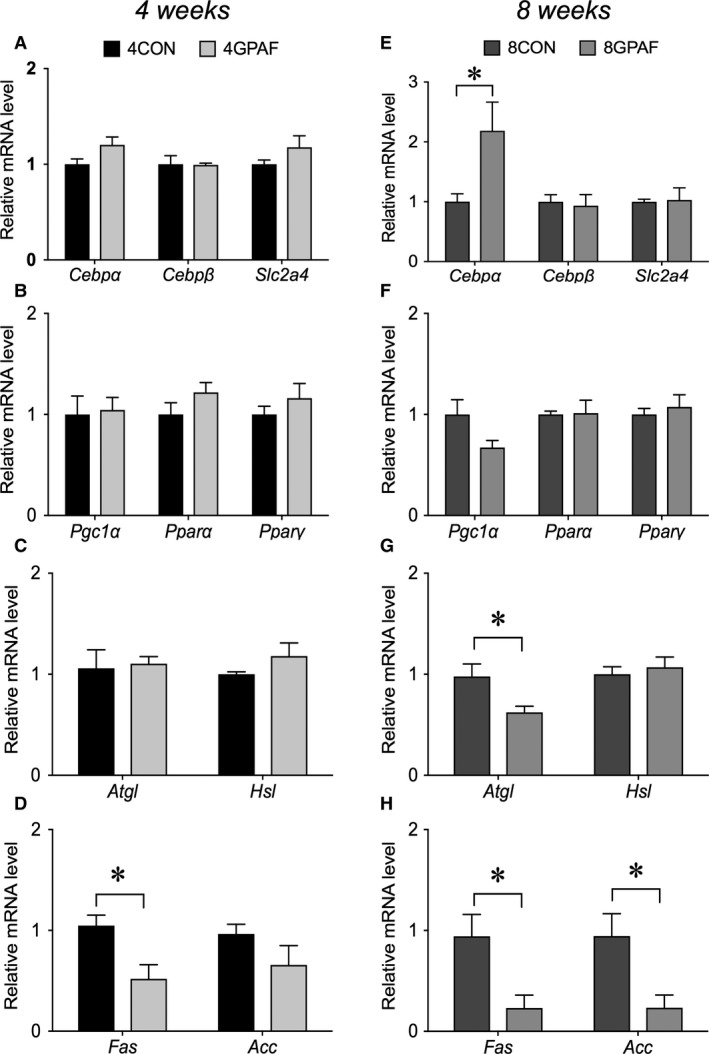
Alteration in the mRNA expression of genes related to adipogenesis and fat metabolism in BAT: mRNA levels were normalized to those of Rps18, and the values for *β*‐GPA‐fed mice were related to the value for each respective control (set to 1). (A, E) Expression of Cebp*α*, Cebp*β*, and Slc2a4 mRNA at week 4 (A) and 8 (E) (B, F) Expression of Pgc1*α*, Ppar*α*, and Ppar*γ* mRNA at week 4 (B) and 8 (F) (C, G) Expression of Atgl and Hsl mRNA at week 4 (C) and 8 (G) (D, H) Expression of Fas and Acc mRNA at week 4 (D) and 8 (H); values are the means ± SEM; *significant difference, *P* < 0.05. Abbreviations are as defined in the legends of Table 1 and Figure 1.

**Figure 7:**
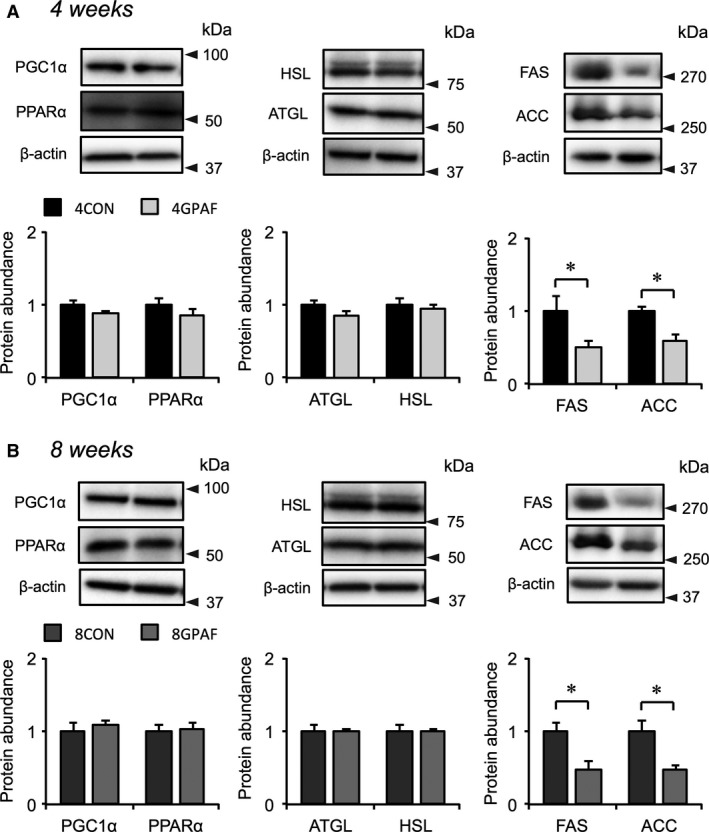
Alterations in the protein expression of PGC‐1*α*, PPAR *α*, ATGL, HSL, FAS, and ACC in BAT at week 4 (A) and 8 (B). The levels of protein expression were normalized to that of *β*‐actin, and the values for *β*‐GPA‐fed mice were related to the value for each respective control (set to 1). Representative western blotting bands are shown above the corresponding bars. Values are the means ± SEM; *significant difference, *P* < 0.05. Abbreviations are as defined in the legends of Table 1 and Figure 1.

We additionally examined the effects of *β*‐GPA feeding on the expression of the same genes and protein in BAT as in eWAT. In BAT, the expression of *Cebpα* was significantly higher in 8GPAF than in 8CON (Fig. 6E). However, the mRNA expression of *Atgl*,* Fas*, and *Acc* was significantly lower in 8GPAF than in 8CON (Fig. 6G and H). At the 4‐week time point of *β*‐GPA feeding, only *Fas* mRNA expression was lower in 4GPAF than in 4CON (Fig. 6D). There were no significant differences in the expression of the other genes between the *β*‐GPA‐fed and control groups (Fig. 6A–H). As shown in Figure 7A and B, there was no significant difference in the protein expression of PGC‐1*α*, PPAR*α*, ATGL, and HSL between the *β*‐GPA‐fed and control groups, whereas that of FAS and ACC was significantly lower in 8GPAF than in 8CON (Fig. 7A and B).

**Figure 8:**
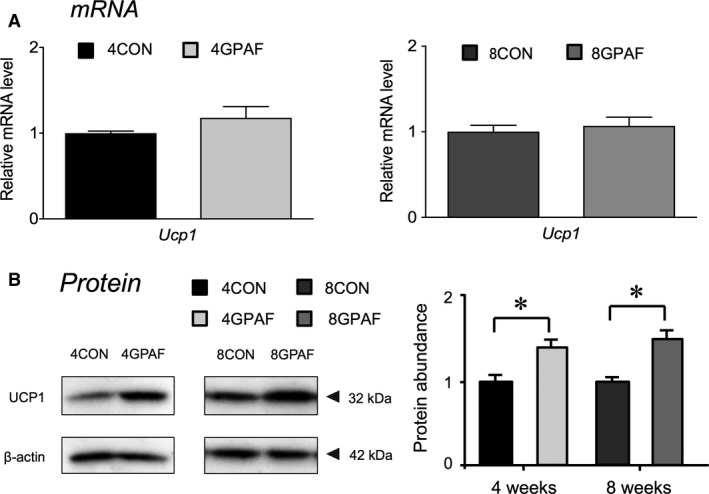
Alterations in the mRNA (A) and protein (B) expression of uncoupling protein 1 (UCP1) in BAT at weeks 4 and 8. The levels of protein expression were normalized to that of *β*‐actin, and the values for *β*‐GPA‐fed mice were related to the value for each respective control (set to 1). Representative western blotting bands are shown above the corresponding bars. Values are the means ± SEM. *significant difference, *P* < 0.05. Abbreviations, except for UCP1, are as defined in the legends of Table 1 and Figure 1.

UCP1 protein expression was more abundant in 4GPAF and 8GPAF than in 4CON and 8CON, respectively, without significant difference in mRNA expression between the groups (Fig. 8).

## Discussion

The current study showed that both 4‐week and 8‐week feeding of *β*‐GPA from 3 weeks of age inhibited the growth‐associated increase in gastrocnemius muscle (Fig. 1D) and eWAT (Fig. 1E) weights. The effects of 4‐week feeding of *β*‐GPA on serum myostatin level and the protein expression of PGC‐1*α* and PPAR*α*, were different from those found after 8‐week feeding of *β*‐GPA in young juvenile mice. These results indicate that the mechanisms involved in *β*‐GPA‐induced inhibition of growth‐associated increase in eWAT weight differ between early and late phases during long‐term feeding of *β*‐GPA in young juvenile mice.

Myostatin is expressed predominantly in skeletal muscle (Mcpherron et al. 1997). Therefore, the increased serum myostatin level in 4GPAF group indicates that *β*‐GPA feeding promoted the production of myostatin in the skeletal muscle of young juvenile mice. Microarray analysis data, which are unpublished results in our laboratory, showed that the expression of *Mstn* in skeletal muscle was 16% higher in 4GPAF than in 4CON, but lower by about 70% in 8GPAF than in 8CON (data not shown). This finding partially supports the changes in serum myostatin level during the *β*‐GPA feeding period shown in Figure 2A. Incubation of C2C12 myotubes with *β*‐GPA for 24 h significantly promoted the gene expression of *Mstn* (Fig. 2B), strongly suggesting that *β*‐GPA directly acts on skeletal muscle cells and stimulates the production of myostatin during its short‐term exposure. However, we could not examine the long‐term effect of *β*‐GPA on *Mstn* expression because C2C12 myotubes are degraded within several days once fully differentiated. Therefore, it remains unclear as to why the serum level of myostatin in 8GPAF was the same as that in 8CON; further work is required to elucidate this.

Incubation of 3T3‐L1 cells with recombinant myostatin protein resulted in the downregulation of gene expression involved in adipogenesis during differentiation (Fig. 3); this corresponded to previous studies showing that myostatin inhibited the adipogenic differentiation of white preadipocytes (Li et al. 2011), brown preadipocytes (Kim et al. 2012) and 3T3‐L1 cells (Rebbapragada et al. 2003). However, even though serum myostatin levels increased at 4 weeks (Fig. 2A), the expression of the adipogenic marker genes, *Cebpα*,* Cebpβ* and *Pparγ*, was not decreased in eWAT (Fig. 4A, B, E and F). This discrepancy in the gene expression between in vivo and in vitro experiments may be attributed to the small population of preadipocytes in eWAT. As the proportion of preadipocytes relative to total cells derived from WAT was at most 5–8% (Tchoukalova et al. 2004), the alterations in adipogenic gene expression in preadipocytes may be masked in whole eWAT.

The previous in vitro study reported that fat accumulation in 3T3‐L1 cells is also diminished by myostatin via inhibition of the expression of critical lipogenic enzymes and promotion of the expression of lipolytic enzymes (Zhu et al. 2015). However, contrary to the present hypothesis, mRNA and protein expression of lipolytic and lipogenic enzymes examined did not always change in concert with the changes in serum myostatin levels. The reason for this discrepancy between the previous finding (Zhu et al. 2015) and the present study remains unknown at present. However, the data shown in Figures 5 and 6 reveal a novel finding: alterations in fat oxidation and energy expenditure in both eWAT and BAT may be linked with the reduced mass of eWAT in response to *β*‐GPA‐induced decreases in the intracellular contents of both creatine and phosphocreatine (Ohira et al. 2003; Oudman et al. 2013).

As shown in Figure 5A, the protein expression of both PGC‐1*α* and PPAR*α* was significantly higher in eWAT of 4GPAF than in that of 4CON, but significantly reduced in 8GPAF compared to that in 8CON. PGC‐1*α* and PPAR*α* are the master regulators of mitochondrial biogenesis (Wu et al. 1999; Arany 2008; Rodgers et al. 2008) and fatty acid oxidation (Mandard et al. 2004; Ribet et al. 2010), respectively. Overexpression of PGC‐1*α* protein upregulates the expression of mitochondrial enzymes involved in fatty acid oxidation and promotes palmitate oxidation, which is well known to be a good index of fatty acid oxidation (Tiraby et al. 2003). It has additionally been reported that the expression of *Pgc1a* mRNA in WAT is inversely correlated with the WAT weight under physiological conditions (Sutherland et al. 2009) and that PPAR*α* activation increased transcription of genes involved in the mitochondrial uptake of fatty acid and *β*‐oxidation in human white adipocytes (Ribet et al. 2010). Finally, Vega et al. (2000) indicated that PPAR*α* and PGC‐1*α* cooperatively increase cellular palmitate oxidation rates in 3T3‐L1 cells. Accordingly, the enhanced expression of both PGC‐1*α* and PPAR*α* proteins may be indicative of the increased fatty acid oxidation in eWAT at the 4‐week time point during *β*‐GPA feeding. In sharp contrast, the potential for fatty acid oxidation may have been blunted in the *β*‐GPA‐fed group at 8 weeks, as the expression of both PGC‐1*α* and PPAR*α* was significantly lower in 8GPAF eWAT than in 8CON eWAT (Fig. 5B).

However, higher UCP1 protein levels were observed in BAT of 8GPAF and 4GPAF compared with that of 8CON and 4CON, respectively, (Fig. 8). The increases in UCP1 protein levels in both *β*‐GPA‐fed groups, together with the marked decreases in the protein expression of lipogenic enzymes FAS and ACC (Fig. 7A and B), indicate that fatty acid utilization may be enhanced throughout the feeding period in BAT. UCP1, which is abundantly expressed in BAT, is responsible for thermogenesis and fatty acid oxidation by dissipating the protein gradient produced by mitochondrial oxidation (Ricquier and Bouillaud 2000; Green et al. 2004; Poher et al. 2015). Therefore, *β*‐GPA feeding is expected to upregulate basal metabolism by enhancing fatty acid oxidation in eWAT and BAT, as in the case of skeletal muscle (Tanaka et al. 1997). Indeed, we have previously demonstrated increased resting metabolic rate and decreased respiratory quotient in response to *β*‐GPA feeding, along with the increased activity of enzymes involved in fatty acid oxidation, in rat skeletal muscle (Tanaka et al. 1997).

Taken together, the present findings show that both the increased UCP1 protein level in BAT and the enhanced fatty acid oxidation in eWAT may inhibit the growth‐dependent increase in fat mass in a synergistic manner until week 4. However, at 8 weeks of *β*‐GPA feeding, eWAT may exhibit compensatory deterioration of fatty acid oxidation to inhibit further reduction in eWAT mass. Supporting this scenario, a smaller difference in eWAT weight was found between 8GPAF and 8CON (37%) compared with that between 4GPAF and 4CON (59%) (Fig. 1E).

Here, we examined the effects of *β*‐GPA feeding on changes in protein expression, in both WAT and BAT. As indicated by the results, however, the changes in protein expression did not always correspond to those in mRNA expression. This suggests that *β*‐GPA feeding modifies post‐transcriptional regulation of the molecules tested, together with transcriptional regulation. In addition, it still remains unclear how long‐term *β*‐GPA feeding has to be performed to cause the differential expressions of PGC1*α* and PPAR*α* protein to the early and the later phase of feeding period in eWAT, as well as in the case of serum myostatin levels. However, it is speculated that this phenomenon may be related to the agonistic effect of *β*‐GPA on AMP‐activated protein kinase (AMPK). *β*‐GPA feeding enhanced the phosphorylation level of AMPK in gastrocnemius muscle of young mouse pups at week 2, and the residual effects of AMPK activation remained at least until week 4 but not week 8 (Baumgarner et al. 2015). Because AMPK enhances myostatin expression in C2C12 myotubes in vitro (Das et al. 2012), it can be speculated that the differential response of serum myostatin to 4‐ and 8‐week *β*‐GPA feeding might depend on the changes in AMPK activity within skeletal muscle. Given that a similar change in AMPK activity may be induced in eWAT, it is likely that the increased expressions of PGC‐1*α* and PPAR*α* protein may be related to a possible increase in AMPK activity in eWAT at the 4‐week time point of *β*‐GPA feeding. The phosphorylation of PPAR*α* and the protein level of PGC‐1*α* are upregulated by an AMPK‐dependent mechanism in adipocytes (Cheng et al. 2015), and AMPK regulates PGC‐1*α* expression and mitochondrial enzymes in eWAT (Wan et al. 2014). However, although it is reported that AMPK modifies the expression of ATGL and HSL protein (Ahmadian et al. 2011; Zhou et al. 2011), the expression levels of these proteins did not change during *β*‐GPA feeding period. Thus, further studies are required to establish an energy sensing network constituted by metabolic sensors including AMPK during the long‐term feeding of *β*‐GPA.

## Conclusion

This study indicates that the inhibition of creatine action by *β*‐GPA feeding for 4 weeks, but not for 8 weeks, increased serum myostatin levels in young juvenile mice. Similarly, the protein expression of PGC‐1*α* and PPAR*α* was upregulated in the eWAT of *β*‐GPA‐fed mice at week 4, but downregulated at week 8. On the other hand, UCP1 protein levels in BAT were enhanced throughout the *β*‐GPA‐feeding period. The inhibitory effect of *β*‐GPA feeding on eWAT and body weight tended to be evident at the early stage. Our findings highlight different mechanisms underlying the inhibition of growth‐dependent normal increases in WAT mass by *β*‐GPA feeding between early and late phases. A more comprehensive understanding of the roles of creatine in controlling body composition would facilitate the development of therapeutic countermeasures against obesity at the growth stage.

## Conflict of Interest

None declared.
